# Ginsenoside compound K sensitizes human colon cancer cells to TRAIL-induced apoptosis via autophagy-dependent and -independent DR5 upregulation

**DOI:** 10.1038/cddis.2016.234

**Published:** 2016-08-11

**Authors:** Lei Chen, Yue Meng, Qi Sun, Zhongyu Zhang, Xiaoqing Guo, Xiaotong Sheng, Guihua Tai, Hairong Cheng, Yifa Zhou

**Affiliations:** 1 Jilin Province Key Laboratory on Chemistry and Biology of Changbai Mountain Natural Drugs, School of Life Sciences, Northeast Normal University, Changchun, China

## Abstract

Tumor necrosis factor (TNF)-related apoptosis-inducing ligand (TRAIL) is a potent cancer cell-specific apoptosis-inducing cytokine with little toxicity to most normal cells. However, acquired resistance of cancer cells to TRAIL is a roadblock. Agents that can either potentiate the effect of TRAIL or overcome resistance to TRAIL are urgently needed. This article reports that ginsenoside compound K (CK) potentiates TRAIL-induced apoptosis in HCT116 colon cancer cells and sensitizes TRAIL-resistant colon cancer HT-29 cells to TRAIL. On a cellular mechanistic level, CK downregulated cell survival proteins including Mcl-1, Bcl-2, surviving, X-linked inhibitor of apoptosis protein and Fas-associated death domain-like IL-1-converting enzyme-inhibitory protein, upregulated cell pro-apoptotic proteins including Bax, tBid and cytochrome c, and induced the cell surface expression of TRAIL death receptor DR5. Reduction of DR5 levels by siRNAs significantly decreases CK- and TRAIL-mediated apoptosis. Importantly, our results indicate, for the first time, that DR5 upregulation is mediated by autophagy, as blockade of CK-induced autophagy by 3-MA, LY294002 or Atg7 siRNAs substantially decreases DR5 upregulation and reduces the synergistic effect. Furthermore, CK-stimulated autophagy is mediated by the reactive oxygen species–c-Jun NH2-terminal kinase pathway. Moreover, we found that p53 and the C/EBP homologous (CHOP) protein is also required for DR5 upregulation but not related with autophagy. Our findings contribute significantly to the understanding of the mechanism accounted for the synergistic anticancer activity of CK and TRAIL, and showed a novel mechanism related with DR5 upregulation.

Tumor necrosis factor (TNF)-related apoptosis-inducing ligand (TRAIL), a member of the TNF cytokine family, is a potent cancer cell-specific apoptosis-inducing agent that exhibits little or no effect on normal tissues.^1, 2, 3^ TRAIL can bind to five distinct type I transmembrane receptors, two of which are death receptors, DR4 (TRAIL-R1) and DR5 (TRAIL-R2), and three of which are decoy receptors, DcR1 (TRAIL-R3), DcR2 (TRAIL-R4) and osteoprotegerin. Each of DRs contains a cytoplasmic functional death domain.^4, 5, 6^ Following engagement with the DRs, TRAIL triggers cell death via both extrinsic and intrinsic apoptosis pathways.^7^ As a result of its selectivity toward tumor cells, both TRAIL and TRAIL-R agonistic antibodies (mapatumumab and lexatumumab) against its receptors are currently in clinical trials for treatment against cancer.^8, 9, 10^

Although TRAIL has shown efficacy in a phase 2 clinical trial, development of resistance to TRAIL by tumor cells is a major roadblock. Numerous mechanisms have been identified by which tumor cells develop resistance to TRAIL. Mechanisms of resistance include the downregulation of DR4 and DR5 expression, upregulation of decoy receptors, the overexpression of the caspase-8 inhibitor, Fas-associated death domain-like IL-1-converting enzyme-inhibitory protein (cFLIP), the hyper-methylation of caspase-8, the overexpression of anti-apoptotic proteins, loss of pro-apoptotic proteins, the overexpression of the inhibitor of apoptosis protein (IAP) family members, and the activation of the PI3K/AKT and NF-kB signaling pathways.^11, 12, 13, 14, 15, 16, 17^ Therefore, the effectiveness of TRAIL and TRAIL-R agonistic antibodies as monotherapies may be limited because of the development of resistance, and agents that can enhance TRAIL-induced apoptosis and sensitize resistant cancer cells to TRAIL are urgently needed.^18, 19^

Natural products have had a profound role in the discovery of cancer drugs over the years. Ginseng has been used for centuries all over the world as a panacea that promotes longevity.^20^ Ginsenosides are the major active ingredients of ginseng. Our research group has screened approximately 20 ginsenosides including the protopanaxadiol-type ginsenosides (Rb1, Rb2, Rc, Rd, F2, Rg3, Rh2, CO, CY, CMc1, CMc and CK), the protopanaxatriol-type ginsenosides (Re, Rg1, Rg2, Rh1 and F1) and gypenoside (XVII and LXXV) for enhancing TRAIL-induced apoptosis or sensitizing resistant cancer cells to TRAIL. The results showed that ginsenoside compound K (CK) and TRAIL could function cooperatively against colon cancer. CK was identified as a major ginsenoside metabolite in urine and blood.^21^ It has been reported that CK enhances gamma ray-induced apoptosis via the generation of reactive oxygen species (ROS) and the disruption of the mitochondrial membrane in human lung cancer cells.^22^ CK also induces apoptosis in MCF-7 human breast cancer cells via ROS generation and the modulation of AMP-activated protein kinase signaling.^23^ In human colon cancer cells, CK induces autophagy and apoptosis via the generation of ROS and the activation of c-Jun NH2-terminal kinase (JNK).^24, 25^ In this study, we reported the possible mechanisms underlying the cooperative induction of apoptosis by the CK and TRAIL combination.

## Results

### CK enhanced TRAIL-induced apoptosis in HCT116 cells

**Figure 1:**
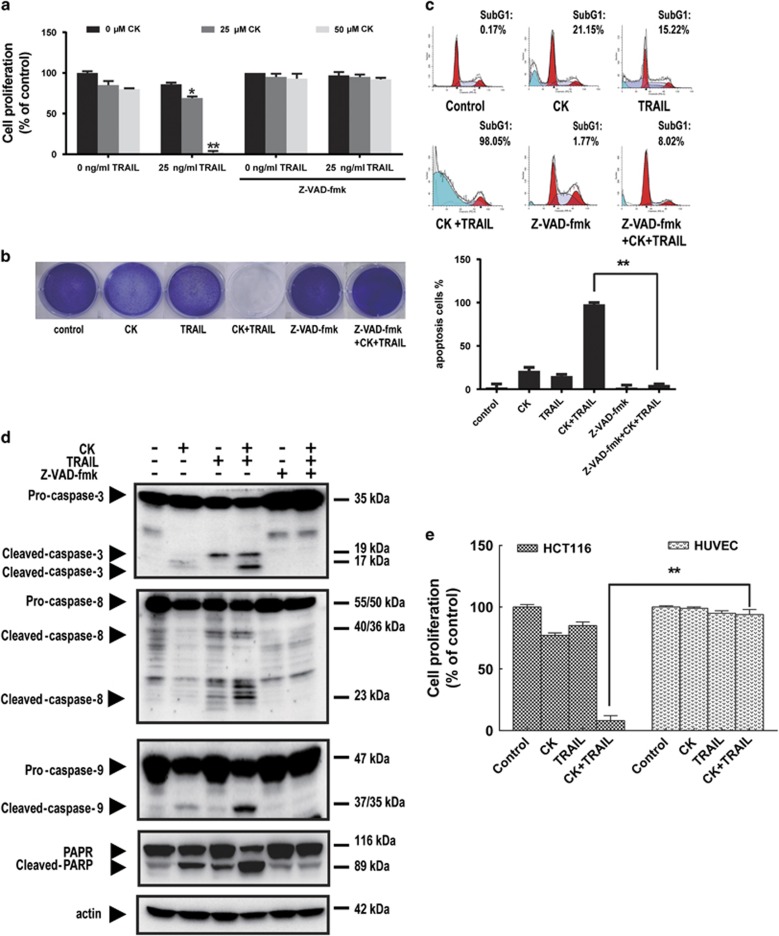
CK enhanced TRAIL-induced apoptosis in HCT116 cells. HCT116 cells or HUVECs were pretreated with or without 20 *μ*M z-VAD-fmk for 1 h, and then treated with or without indicated doses of CK for 24 h and co-treated with or without TRAIL (25 ng/ml) for 24 h. Cell viability was analyzed using WST-1 assay (**a** and **e**) and crystal violet staining assay (**b**). Cell apoptosis (as indicated by sub-G1 DNA content to the left of the G1 peak) was tested by flow cytometry followed by PI staining (**c**), the processing of the caspases was tested by western blot followed by pretreated with or without 20 *μ*M z-VAD-fmk for 1 h, and then treated with CK (25 *μ*M; 48 h) and TRAIL (25 ng/ml; 24 h). Actin was used as a protein loading control (**d**). Error bars in (**a**), (**c**) and (**e**) represent the S.D. (*N*=3 independent experiments). *P<0.05, ** *P*<0.01 compared with DMSO group (Student's *t*-test, two tailed)

**Figure 2:**
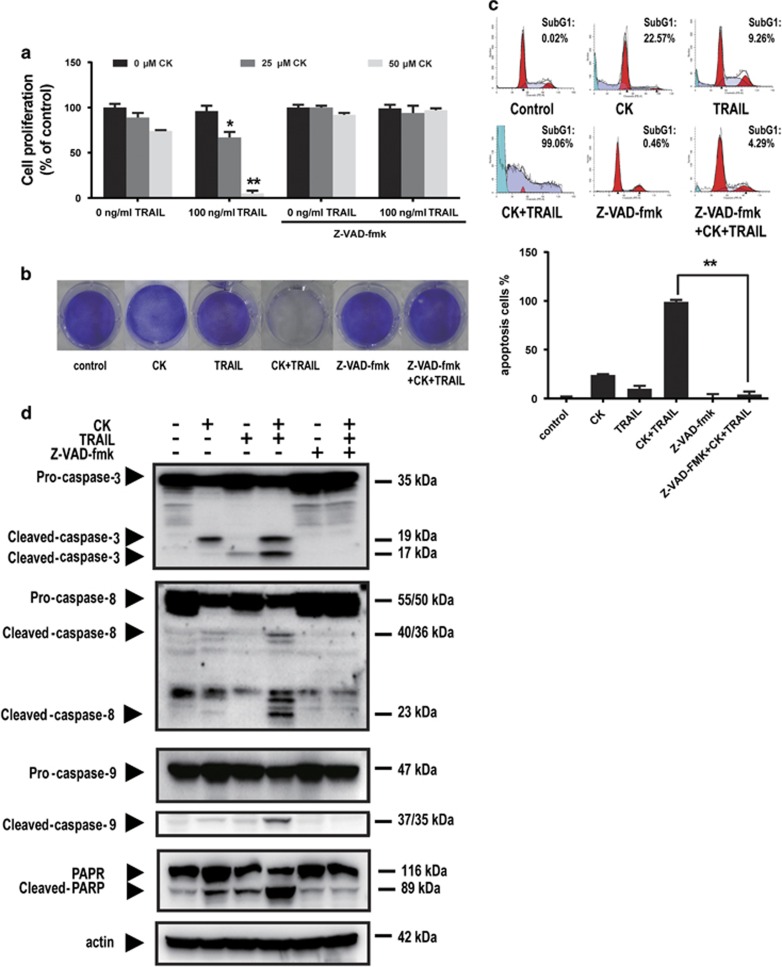
CK sensitized TRAIL-resistant HT-29 cells to TRAIL. HT-29 cells were pretreated with or without 20 *μ*M z-VAD-fmk for 1 h, then treated with indicated doses of CK for 24 h and co-treated with or without TRAIL (100 ng/ml) for 24 h. Cell viability was analyzed using the WST-1 assay (**a**) and crystal violet staining assay (**b**). Cell apoptosis (as indicated by sub-G1 DNA content to the left of the G1 peak) was tested by flow cytometry followed by PI staining (**c**), the processing of the caspases was tested by western blot followed by pretreated with or without 20 *μ*M z-VAD-fmk for 1 h, and then treated with CK (25 *μ*M; 48 h) and TRAIL (100 ng/ml; 24 h). Actin was used as a protein loading control (**d**). Error bars in (**a**) and (**c**) represent the S.D. (*N*=3 independent experiments). *P<0.05, **P<0.01 compared with DMSO group (Student's *t*-test, two tailed)

To investigate whether CK could synergize with TRAIL to inhibit the colon cancer cell viability, a panel of TRAIL-sensitive colon cancer cell lines including HCT116 (Figure 1a), colo205, DLD-1, SW480 (Supplementary Figure 1a) cells and TRAIL-resistant HT-29 (Figure 2a) cells were tested. The results showed that the combination regime exerted robust synergistic effect on these cell lines.

HCT116 cells were moderately sensitive to 25 *μ*M or 50 *μ*M CK and only slightly sensitive to 25 ng/ml TRAIL. However, when the cells were pretreated with 50 *μ*M CK for 24 h followed by co-treated with 25 ng/ml of TRAIL for 24 h, cells underwent marked cell death as evidenced by WST-1 assay (Figure 1a).

The effect of CK on TRAIL-induced suppression of cell viability was confirmed by the crystal violet staining assay (Figure 1b). The results of a flow cytometry analysis for apoptosis showed that CK or TRAIL alone induced 21.15 or 15.22% apoptosis, respectively. The combination treatment with CK and TRAIL enhanced apoptosis to 98.05% (Figure 1c). A quantitative data with mean and S.D. of three independent experiments showed similar results (Figure 1c). As single agents, CK and TRAIL induced low levels of caspase-8, -9 and -3 and poly(ADP-ribose) polymerase cleavage, whereas the combination induced profoundly higher processing of these proteins (Figure 1d). Pretreatment with the cell-permeable pan caspase inhibitor, z-VAD-fmk, profoundly blocked the synergistic effect by this treatment (Figures 1a and d), indicating that CK enhanced TRAIL-induced apoptosis of HCT116 cells in a caspase-dependent manner. But in normal human umbilical vein endothelial cells (HUVECs), the combination did not show any synergy (Figure 1e).

### CK sensitized TRAIL-resistant cells to TRAIL-induced apoptosis

We also investigated the synergistic effect of CK and TRAIL in TRAIL-resistant HT-29 cancer. The WST-1 assay results showed that HT-29 cells were moderately sensitive to 25 *μ*M or 50 *μ*M CK and resistant to 100 ng/ml TRAIL. However, the combination of CK and TRAIL significantly suppressed cell viability (Figure 2a). Similar results were obtained with crystal violet staining (Figure 2b). The results of flow cytometry also revealed that CK or TRAIL treatment alone induced 22.57 or 9.26% apoptosis, respectively. The combination treatment with CK and TRAIL enhanced apoptosis to 99.06% (Figure 2c). A quantitative data with mean and S.D. of three independent experiments showed similar results (Figure 2c). Consistent with these findings, the combination treatment more effectively initiated caspase-8, -9 and -3 processing (Figure 2d). Pretreatment with z-VAD-fmk also effectively blocked the synergistic effect induced by this treatment (Figures 2a and d), indicating the caspase-dependent mechanism. In all, our results indicated that CK could enhance TRAIL-induced apoptosis in HCT116 cells and TRAIL-resistant HT-29 cells but not in HUVECs, indicating the sensitization could be limited to cancer cells.

### CK suppressed expression of cell survival proteins and induced expression of pro-apoptotic proteins

**Figure 3:**
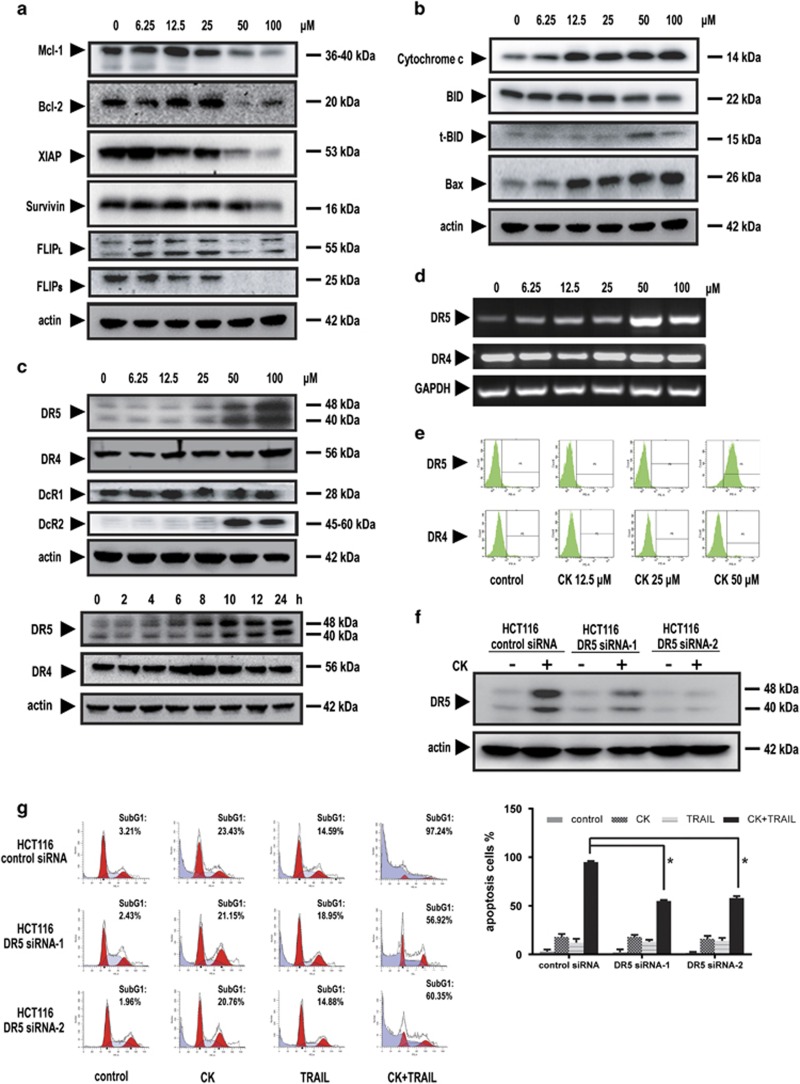
DR5 had an important role in the synergistic effect of CK and TRAIL. HCT116 cells were treated with CK at the indicated doses for 24 h. The anti-apoptotic (**a**), pro-apoptotic proteins (**b**) and DR4/ DR5 proteins (**c**) were analyzed by western blotting. Actin was used as a protein loading control. The DR4 and DR5 mRNA levels were analyzed by RT-PCR after HCT116 cells were treated with CK at the indicated doses for 24 h. GAPDH was used as an internal control to show equal RNA loading (**d**). The cell surface DR4 and DR5 expression was tested by flow cytometry followed by CK (50 *μ*M; 24 h) treatment (**e**). HCT116 cells were transfected with control siRNA or DR5 siRNAs. After treatment with CK for 24 h, whole-cell extracts were prepared and analyzed by western blotting (**f**). The resultant cells were exposed to 50 *μ*M CK for 24 h and then co-treated with or without 25 ng/ml TRAIL for 24 h. Cells were stained with PI and cell death (as indicated by sub-G1 DNA content to the left of the G1 peak) was measured by FACS (**g**). Error bars in (**g**) represent the S.D. (*N*=3 independent experiments). *P<0.05, compared with DMSO group (Student's *t*-test, two tailed)

For mechanistic insight into CK enhancement to TRAIL-induced apoptosis, we quantified multiple extrinsic and intrinsic cell death pathway components that could be affected by CK. The results showed that the caspase proteins including caspase-3, -8, -9 were cleaved (Supplementary Figure 3), the expression of anti-apoptotic proteins including Mcl-1, Bcl-2, X-linked inhibitor of apoptosis protein (XIAP), survivin and cFLIP were suppressed (Figure 3a), the expression of anti-apoptotic proteins including Bax, tBid, cytochrome *c* were upregulated (Figure 3b), which may account, at least in part, for the synergistic effect.

### DR5 induction by CK was required for TRAIL-induced apoptosis

Decreased expression of TRAIL receptors DR4 and DR5 and/or upregulation of the decoy receptors DcR1 and DcR2 account for TRAIL resistance in certain cancer cell lines. We examined the effect of CK on the expression of TRAIL DRs in this study. The results showed that DcR1 and DR4 were not impacted by CK in HCT116 cells (Figure 3c). DR5 and DcR2 were upregulated upon CK treatment in a dose-dependent manner (Figure 3c). However, the upregulation of DR5 was predominant compared with DcR2. Consistent with the protein changes, CK treatment increased DR5 transcription and cell surface expression (Figures 3d and e).

We hypothesized that DR5 had a critical role in the synergistic effect. Kinetic analysis showed that synergistic effect occurred about 12 h after CK pretreatment (Supplementary Figure 1b), well after DR5 upregulation that occurred after treatment for 8 h (Figure 3c). In support of the hypothesis that upregulation of DR5 is indeed mediating the observed cooperative effect of CK and TRAIL. To substantiate this, two specific siRNAs was applied to silence DR5 expression (Figure 3f). The flow cytometry analysis revealed that the silencing of DR5 reduced the percentage of cell apoptosis from 97.24 to 56.92% or 60.35%, respectively (Figure 3g), providing further evidence that DR5 indeed had a crucial role in enhancing the effect of CK on TRAIL-induced apoptosis. A quantitative data with mean and S.D. of three independent experiments showed similar results (Figure 3g).

Other ginsenosides including 20(S)-protopanaxadiol ginsenoside Rd, Rg1 and Rg2 were also tested at similar concentrations. Ginsenoside Rd and Rg1 failed to induce DR5 upregulation (Supplementary Figure 2a), suggesting the DR5 modulation specificity of CK.

We are particularly interested in the mechanism accounted for the upregulation of DR5 response to CK. The reason is that agonistic TRAIL-R antibodies that selectively target DR4 and DR5 induce apoptosis by the same mechanism as TRAIL. However, the stability of TRAIL-R antibodies *in vivo* makes them attractive agents for use in humans, and several clinical trials investigating TRAIL-R antibodies in solid and hematological tumors have been complete initiated. The upregulation of DR5 provided a possibility of the combination of CK and TRAIL-R antibodies. We next sought to identify pathways and transcription factors involved in CK-induced DR5 upregulation.

### Autophagy-mediated DR5 upregulation

**Figure 4:**
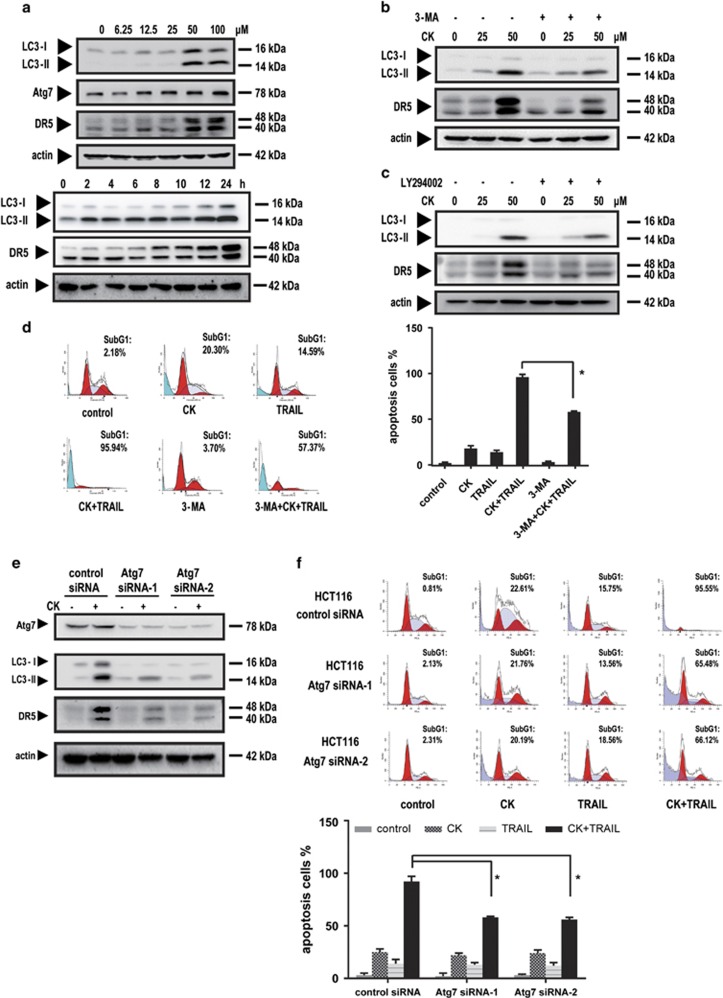
DR5 upregulation by CK was related to autophagy. HCT116 cells were pretreated with (**b**) or without (**a**) the autophagy inhibitor 3-MA, LY294002 (**c**) or transfected with Atg7 siRNA (**e**) and then exposed to CK at the doses indicated for 24 h. Whole-cell extracts were prepared and analyzed by western blotting using protein-specific antibodies. HCT116 cells were pretreated with 3-MA for 1 h (**d**) or transfected with Atg7 siRNA (**f**) and then treated with CK and/or TRAIL for 24 h. Cell death (as indicated by sub-G1 DNA content to the left of the G1 peak) was measured by FACS (**d** and **f**). Error bars in (**d** and **f**) represent the S.D. (*N*=3 independent experiments). *P<0.05 compared with DMSO group (Student's *t-*test, two tailed)

It has been reported that CK could induce autophagy in human colon cancer cells.^25^ The accumulation of autophagosomes in TRAIL-resistant breast cancer cells induces TRAIL resistance through downregulation of surface expression of DR4 and DR5.^26^ Here we investigated whether autophagy was related with the DR5 induction by CK and potentiation of CK on TRAIL-induced apoptosis. LC3 protein is involved in the formation of autophagosomes, and its turnover from the cytosolic form LC3-I to the lipidated form LC3-II has been widely used as a molecular marker of autophagosomes. Atg7 is a ubiquitin ligase-like protein that is specifically required for autophagy. Our results showed that CK produced a profound increase in the levels of LC3-II and Atg7 in a dose- and time-dependent manner (Figure 4a). Pretreatment with the autophagy inhibitor 3-methyladenine (3-MA) at a concentration of 5 mM (Figure 4b) or LY294002 at a concentration of 25 *μ*M (Figure 4c) inhibited LC3-II expression, indicating the reduction of autophagy. These results are in agreement with previous reports.^25^ The time course results showed DR5 upregulation paralleled the changes of LC3-II (Figure 4a). Pretreatment with 3-MA or LY294002 substantially reduced DR5 expression (Figures 4b and c), indicating the autophagy-dependent manner. Consistently, the 3-MA pretreatment reduced the extent of CK/TRAIL-induced apoptosis from 95.94 to 57.37% (Figure 4d). Severe toxicity of 3-MA limited its use at higher concentrations that may better suppress autophagy and DR5 upregulation. Nevertheless, the synergistic effect were significantly inhibited by the concentration of 3-MA that was used.

To exclude the off-target of the inhibitors, transfection of cells with Atg7 siRNAs were applied. LC3-II accumulation and the subsequent DR5 upregulation was attenuated significantly (Figure 4e). The apoptosis induced by the combined treatment was suppressed from 95.55 to 65.48% or 66.12% in the resultant cells, respectively (Figure 4f).

On the basis of the above results, it can be concluded that autophagy had a critical role in the DR5 upregulation and the synergistic effect of CK and TRAIL. As far as we know, this is the first report relating autophagy and DR5 upregulation, and it may provide a novel strategy for restoring cancer cell sensitivity to TRAIL-induced apoptosis.

### JNK activation had a crucial role in autophagy-mediated DR5 expression

**Figure 5:**
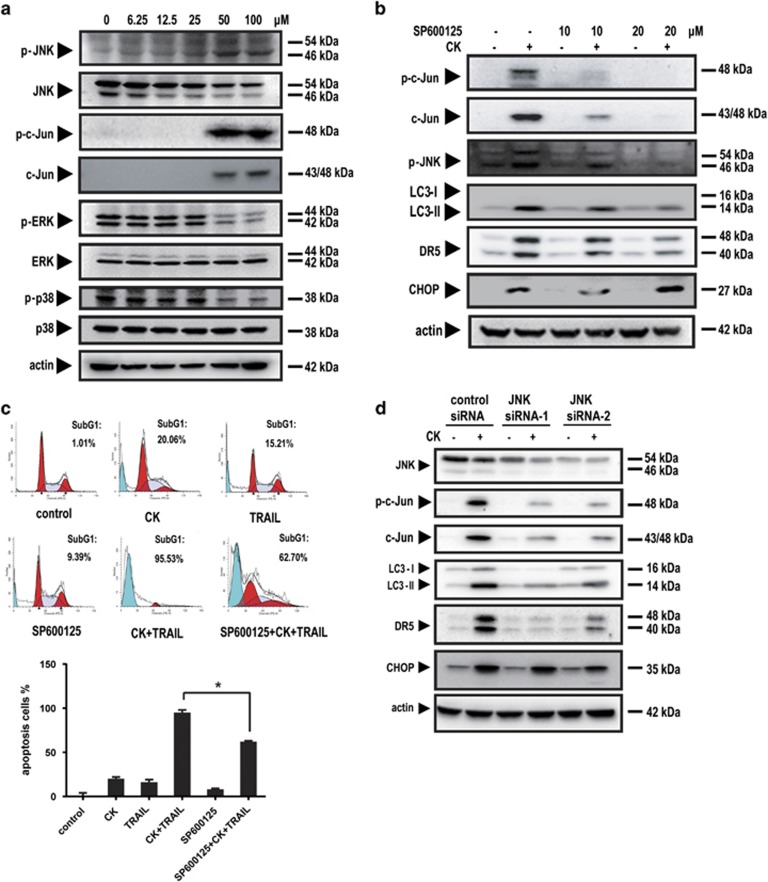
JNK kinase expression had a crucial role in autophagy-mediated DR5 expression. HCT116 cells were pretreated with (**b**) or without (**a**) the JNK inhibitor SP600125 or transfected with JNK siRNAs (**d**) and then exposed to CK at the indicated doses. Whole-cell extracts were prepared and analyzed by western blotting using protein-specific antibodies. HCT116 cells were pretreated with SP600125 for for 1 h and then treated with 50 *μ*M CK for 24 h and then co-treated with or without 25 ng/ml TRAIL for 24 h (**c**). Cell death (as indicated by sub-G1 DNA content to the left of the G1 peak) was measured by FACS. Error bars in (**c**) represent the S.D. (*N*=3 independent experiments). *P<0.05 compared with DMSO group (Student's *t*-test, two tailed)

As mitogen-activated protein kinases (MAPKs), including the extracellular signal-regulated kinases (ERKs), JNKs and p38-MAPKs, can mediate DR5 upregulation,^18, 27, 28^ we first investigated whether these kinases had a role in CK-induced DR5 expression. The results showed that CK increased JNK phosphorylation, but decreased ERK or p38 phosphorylation, in a dose-dependent manner (Figure 5a). Downregulation of ERK and p38 by CK, which may account, at least in part, for the synergistic effect. The c-Jun phosphorylation was substantially induced, confirming the JNK activation upon CK treatment (Figure 5a).

Pretreatment with the JNK inhibitor SP600125 profoundly reduced activation of JNK and the subsequent upregulation of DR5 (Figure 5b), suggesting that JNK activation was related to the induction of DR5. Further, SP600125 also attenuated LC3-II expression (Figure 5b), indicating a JNK–autophagy pathway. Further, the results of the flow cytometry assay showed that SP600125 reduced the extent of apoptosis induced by CK and TRAIL from 95.53 to 62.70% (Figure 5c). From the above results, we can conclude that JNK activation had a crucial role in autophagy-mediated DR5 expression.

To rule out of the off-target effects of SP600125, we further tested the effects in JNK siRNAs transfected cells. The results showed that JNK siRNAs attenuated the effect of LC3-II accumulation and the subsequent DR5 upregulation (Figure 5d).

### ROS had a critical role in JNK–autophagy-mediated DR5 expression

**Figure 6:**
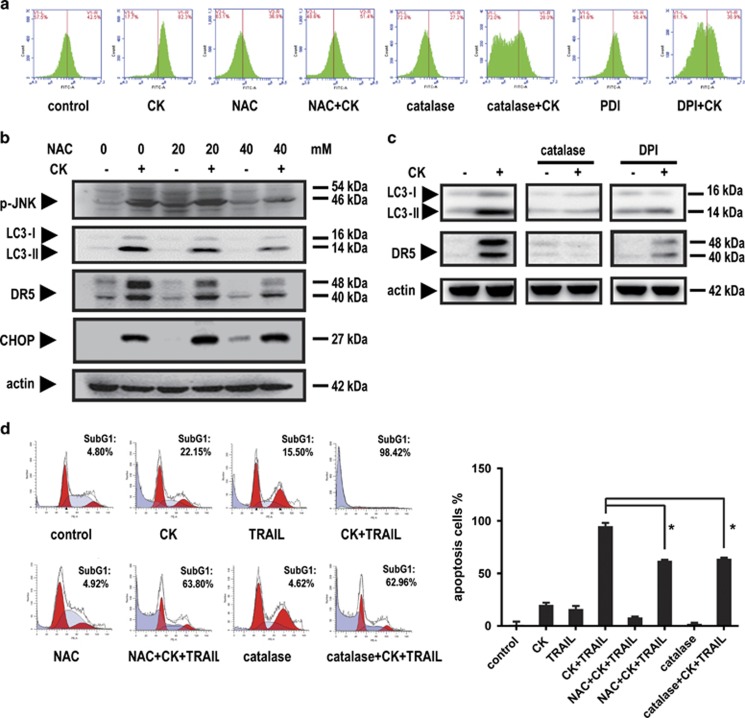
ROS production was involved in autophagy-mediated DR5 expression. HCT116 cells were pretreated with or without 40 mM NAC, 5 *μ*M DPI or 25 mM catalase for 1 h and then treated with or without CK for 18 h. ROS levels were assessed by flow cytometry followed by DCFH-DA staining (**a**). HCT116 cells were pretreated with 40 mM NAC for 1 h and then exposed to 50 μM CK for 24 h. Whole-cell extracts were prepared and analyzed by western blotting (**b**). HCT116 cells were pretreated with catalase and DPI for 1 h and then exposed to 50 μM CK for 24 h. Whole-cell extracts were prepared and analyzed by western blotting (**c**). HCT116 cells were pretreated as in (**b**) and then treated with or without 25 ng/ml TRAIL for 24 h. The cells were stained with PI and analyzed by flow cytometry (**d**). Error bars in (**d**) represent the S.D. (*N*=3 independent experiments). *P<0.05 compared with DMSO group (Student's *t*-test, two tailed)

Previous reports have indicated that DR5 expression can be induced by ROS.^29,30,31^ Here, we sought to determine whether CK-induced DR5 expression was also regulated by ROS. The results showed that CK did stimulate the production of ROS in HCT116 cells in a time-dependent manner (Supplementary Figure 4). The enhanced ROS levels could be attenuated by pretreatment with the ROS scavenger *N*-acetylcysteine (NAC), diphenyleneiodonium chloride (DPI) and catalase (Figure 6a), confirming the participation of ROS. Furthermore, pretreatment of these cells with NAC, DPI or catalase inhibited DR5 upregulation (Figures 6b and c), indicating that ROS production was related to the expression of DR5. Further, pretreating HCT116 cells with 40 mM NAC reduced the level of JNK phosphorylation and the subsequent LC3-II expression (Figure 6b). Consistently, pretreatment of these cells with 40 mM NAC and 25 mM catalase inhibited CK/TRAIL-induced apoptosis from 98.42 to 63.80% or 62.96%, respectively (Figure 6d).

On the basis of these observations, we concluded that CK upregulated DR5 expression via the ROS–JNK–autophagy pathway.

### C/EBP homologous protein (CHOP) was related with DR5 expression

**Figure 7:**
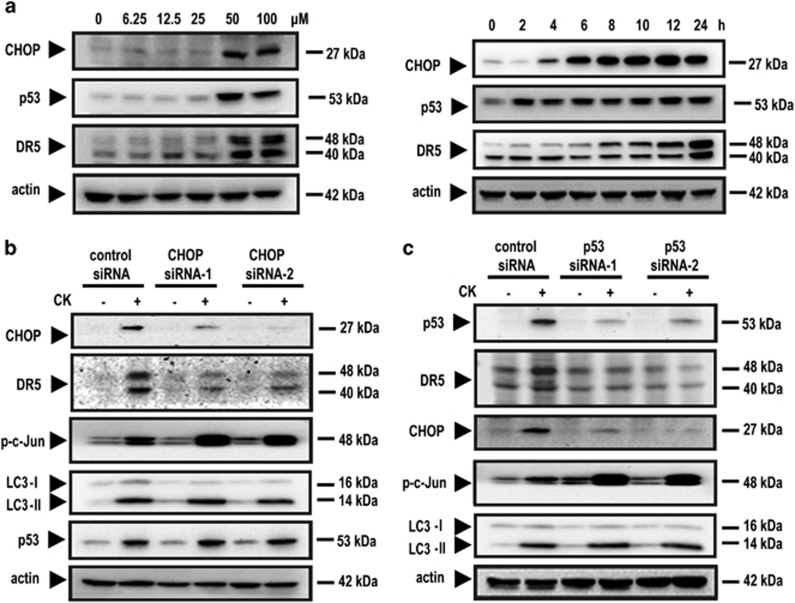
CHOP and p53 influenced upregulation of DR5. HCT116 cells were pretreated with CK at the indicated doses for 24 h or with CK at 50 μM for the times indicated. Whole-cell extracts were prepared and analyzed by western blotting (**a**). HCT116 cells were transfected with control siRNA or CHOP siRNA and then exposed to 50 μM CK for 24 h. Whole-cell extracts were prepared and analyzed by western blotting (**b**). HCT116 cells were transfected with control siRNA or p53 siRNAs (**c**) and were treated with CK at the doses indicated. Whole-cell extracts were then prepared and analyzed by western blotting

So far, our results showed that blocking the ROS–JNK–autophagy pathway only partially attenuated CK-induced upregulation of DR5. This result suggested that ROS–JNK–autophagy was not the only pathway that accounted for the DR5 upregulation upon CK treatment. It has been reported that CHOP could also cause the upregulation of DR5,^32^ we examined the effect of CK on CHOP expression. Our results showed that CK increased the expression of CHOP in a dose- and time-dependent manner (Figure 7a). In the same cell system, we observed that the induction of DR5 paralleled the increase of CHOP, occurred following 8 or 4 h of CK treatment, respectively (Figure 7a). Transfection of cells with CHOP siRNAs substantially reduced DR5 expression (Figure 7b), suggesting that CHOP also had a role in CK-induced DR5 upregulation. In addition, the knockdown of CHOP did not suppressed LC3-II expression, indicating that the CHOP did not influence autophagy.

### The involvement of p53 in CK-induced sensitization to TRAIL

*DR5* is known a p53 target gene. It has been reported that DNA-damaging agents synergized with TRAIL by inducing p53-dependent transcription of DR5. However, p53 mutations commonly arise in colorectal cancer cells. The use of DNA-damaging agents for TRAIL sensitization would likely be ineffective in the absence of wild-type p53.

Therefore, we investigated whether p53 had a role in the DR5 upregulation by CK. The results showed that CK also downregulated DR5 expression in these p53 siRNAs transfected HCT116 cells (Figure 7c), indicating the p53-dependent mechanism. Interestingly, p53 knockdown also downregulated CHOP expression but did not impact LC3-II expression (Figure 7c), suggesting DR5 was also regulated through p53-CHOP pathway, which is not related with autophagy. The combination of CK and TRAIL would be more effective on the human colon cancers with wild-type *p53*.

### A proposed mechanism to account for the effects of CK and TRAIL

To investigate whether p53-CHOP and ROS–JNK–autophagy pathways operated dependently or independently on DR5 upregulation upon CK treatment, we examined the activation of CHOP in JNK siRNA transfected (Figure 5d) or NAC pretreated cells (Figure 6b), as well as c-Jun phosphorylation in CHOP siRNAs and p53 siRNAs transfected cells (Figures 7b and c). All the results suggested that these two pathways function independently of each other.

**Figure 8:**
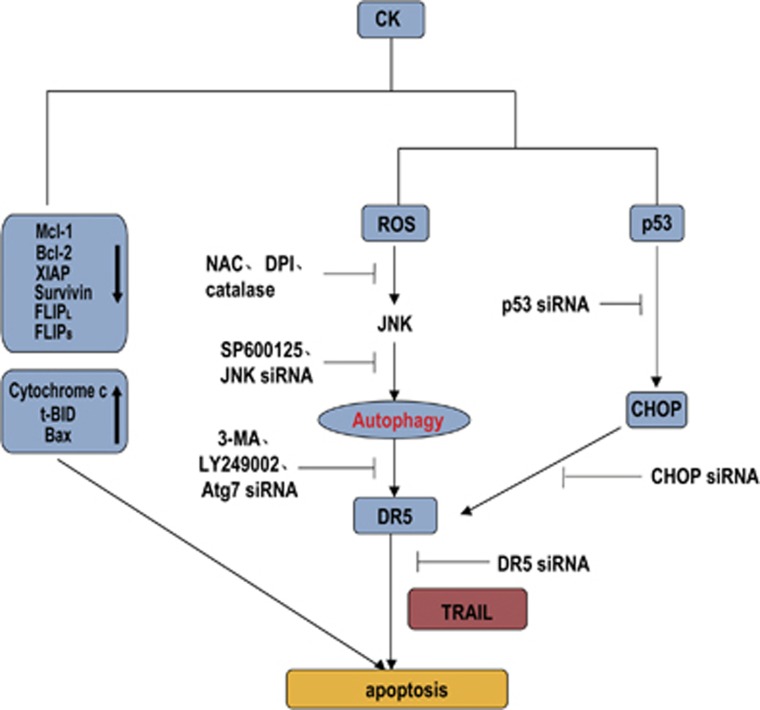
An illustration of a likely mechanism of action through which CK potentiates TRAIL-induced apoptosis

Figure 8 illustrates a likely mechanism of action for the effects of CK on TRAIL-induced apoptosis. Based on our results, we concluded that CK enhanced the pro-apoptotic effect of TRAIL on colon cancer cells by suppressing expression of cell survival protein, inducing expression of pro-apoptotic proteins, autophagy-dependent and autophagy-independent (p53-CHOP pathway) DR5 upregulation. These two pathways could operate independently of the other.

## Discussion

Recombinant soluble TRAIL and agonistic antibodies against its receptors are actively being developed for clinical cancer therapy because of its superior safety profile and high tumor specificity compared with other TNF family members. However, few objective responses have been reported.^8, 9^ It is possible that adjuvant therapies will be required to enhance their effectiveness. Thus, agents that can either potentiate the effect of TRAIL or overcome resistance to it are urgently needed.

Approximately 20 ginsenosides were screened by testing the pro-apoptotic effect combined with TRAIL in human colon cancer cells in our laboratory. Ginsenoside CK was found to enhance TRAIL-induced apoptosis in a panel of TRAIL-sensitive cells including HCT116, colo205, DLD-1 and SW480 cells and TRAIL-resistant HT-29 cells. CK was identified as a major ginsenoside metabolite in urine and blood. Hence, the mechanism of action by which CK sensitized cancer cells to TRAIL-induced apoptosis may be the general mechanism of ginsenosides.

It has been reported that serial blood samples were collected during 36 h after Korean Red Ginseng extract administration by 10 healthy Korean male volunteers to determine plasma concentrations of CK. The mean maximum plasma concentration of CK was 8.35±3.19 ng/ml (13.4±5 mM).^33^ CK sensitized colon cancer cells to TRAIL at 50 *μ*M in this study, a concentration much less than the peak plasma concentrations achieved in volunteer during the test. Hence, the clinical application of CK was as guaranteed.

At a mechanistic level, our results showed that CK downregulated cell survival proteins, including Mcl-1, Bcl-2, XIAP, survivin and cFLIP (Figure 3a), upregulated pro-apoptotic proteins including Bax, tBid, cytochrome *c* (Figure 3b), and upregulated DR5 cell surface expression (Figure 3c) in a dose-dependent manner.

We are particularly interested in the role of DR5 in the synergistic effect and the mechanism accounted for the upregulation of DR5. The reason is that agonistic TRAIL-R antibodies that selectively target DR4 and DR5 are more attractive for use in humans compared with TRAIL, and several clinical trials investigating TRAIL-R antibodies in solid and hematological tumors have been complete initiated. The upregulation of DR5 by CK provided a possibility that the combination of CK and TRAIL-R antibodies.

Our results indicated that DR5 was crucial to the combined effect because gene silencing of DR5 decreased the effect of CK on TRAIL-induced apoptosis. Notably, our results, for the first time, indicated that CK induced DR5 expression via autophagy. Autophagy is an evolutionarily conserved catabolic process that begins with the formation of autophagosomes, which participate in the recycling of cellular components by sequestering damaged organelles and misfolded proteins and targeting them for lysosomal degradation. As a response to anticancer treatments, whether autophagy activation leads to cell survival or cell death remains unclear.^34^ Previous studies have suggested that the induction of autophagy could be a useful therapeutic approach by which to overcome drug resistance of cancers to some therapeutic agents, particularly those that typically induce an apoptotic response.^35, 36^ Here, we found that CK increases LC3-II and Atg7 expression, indicating the onset of autophagy, and pretreatment with 3-MA, LY294002 or Atg7 siRNAs, which inhibits autophagy, reduces DR5 expression. This is the first report of a link between autophagy and DR5 upregulation, and thus it provides a potential therapeutic strategy by which to restore the sensitivity of cancer cells to TRAIL-induced apoptosis.

It has been reported that autophagy regulates key processes associated with TRAIL resistance. However, the molecular mechanisms of autophagy-mediated TRAIL resistance still need to be elucidated.^37, 38^ Recently, TRAIL-resistant breast cancer cells were reported to have a high basal level of autophagosomes that sequestered DR4 and DR5, which may contribute to the deficiency of these molecules on the cell surface.^26^ The disruption of autophagosome structures (e.g., using 3-MA or Atg7 siRNA) restores the surface expression of DR4 and DR5, thus making cells susceptible to TRAIL-induced apoptosis in TRAIL-resistant cancer cells.^26^ In TRAIL-sensitive MDA-MB-23 cells, pretreated with autophagy inhibitor bafilomyin A1 or chloroquine, the accumulation of LC3-II protein is much higher compared with BT474 and AU565 cells. In addition, bafilomycin A1 treatment induced a significant DRs downregulation from surface membrane in a time-dependent manner. The resultant cells became less sensitive to TRAIL-induced apoptosis.^26^ It seems that MDA-MB-23 cells undergo a rapid lysosomal turnover, which explain the lack of basal autophagosomes. Autophagosomes are thought to be efficient carriers of a broad spectrum of cellular antigens, which may include tumor cell surface receptors.

Our results are consistent with their findings. CK induced significant autophagy flux, which accounted for the upregulation of DR5 and the thereby synergistic effect of CK and TRAIL. Although the precise roles of TRAIL and its receptors in endocytosis and turnover remain unclear.

In our study, we found that CK induced autophagy-mediated DR5 expression via the generation of ROS and the activation of JNK. Previous research showed that CK induced autophagy via generation of ROS and activation of JNK,^25^ which is consistent with our findings.

Our data suggested that ROS–JNK activation was not sufficient for CK-induced DR5 expression. The CHOP pathway, which we showed functioned independently of ROS–JNK activation, was also involved in promoting DR5 expression. CHOP, a transcription factor in the C/EBP family, was involved in endoplasmic reticulum stress, including the unfolded protein response. CHOP can bind to members of the C/EBP family to regulate transcriptional activity and enhance AP-1 complex formation. Various stimuli are known to promote CHOP binding to the DR5 promoter and thus upregulate DR5 expression.^39, 40^ Another ginsenoside, Rg3, can similarly induce DR5 expression via the induction of CHOP,^41^ suggesting a similar mechanism of action for ginsenosides in general. Further studies are needed to substantiate this hypothesis.

*DR5* gene is regulated in a p53-dependent and -independent manner during genotoxic and nongenotoxic stress-induced apoptosis.^42, 43^ The role of p53 on the induction of DR5 depends on the nature of the stimulus.^18, 44^ Here we found that p53 had a role in the expression of DR5. It is suggested that the combination are more effective in colorectal cancer cells with wild-type *p53*.

Overall, our results provided the first mechanistic evidence that CK treatment of cancer cells results in sensitization of TRAIL and enhancement of TRAIL-induced apoptosis through autophagy-dependent and -independent (p53-CHOP pathway) DR5 upregulation. Our findings contribute substantially to the understanding of the anticancer activity of CK and warrant further evaluation of the combination of CK and TRAIL as a potential therapeutic regimen against human colon cancer.

## Materials and Methods

### Reagents

A concentration of 200 mM CK (purchased from Chengdu Herbpurify Co., Ltd, Chengdu, China) was prepared in 100% dimethyl sulfoxide, stored in small aliquots at –80 °C and diluted in cell culture medium as needed. His-tagged recombinant human TRAIL vector is a kind gift from Professor Wafik S El-Deiry (Fox Chase Cancer Center, Philadelphia, PA, USA). The protein was produced and purified as described previously.^45^ Dulbecco's modified Eagle's medium: nutrient mixture F-12 (DMEM/F-12) medium, Iscove's modified Dubecco's medium and fetal bovine serum (FBS) were obtained from Gibco (Grand Island, NY, USA). Trypsin and phenylmethylsulfonyl fluoride (PMSF) were obtained from Amersco LLC (Solon, OH, USA). DPI, catalase, PI and WST-1 were purchased from Sigma Aldrich (St. Louis, MO, USA). Incomplete protease inhibitor cocktail tablets were acquired from Roche (Welwyn Garden City, UK). The ECL Plus Western Blotting Detection Kit was purchased from GE Healthcare (Little Chalfont, UK). Penicillin/streptomycin was obtained from the Tina Jin Hao Yang Biological Manufacture Co., Ltd. (Tianjin, China). Antibodies against DR4 and caspase-9 were obtained from Santa Cruz Biotechnology (Santa Cruz, CA, USA). Antibodies against actin and caspase-8 were purchased from BD Biosciences (Franklin Lakes, NJ, USA). Other antibodies were purchased from Cell Signaling (Danvers, MA, USA). Caspase inhibitor and SP600125 and LY294002 were purchased from Beyotime Institute of Biotechnology (Shanghai, China). All of the other reagents were of analytical grade or better.

### Cells and culture conditions

Unless otherwise stated, all of the cell lines were purchased from the American Type Culture Collection (Manassas, VA, USA) and were maintained in the appropriate growth medium supplemented with 10% FBS and penicillin/streptomycin at 37 °C and 5% CO_2_. HUVECs were cultured in DMEM medium.

### Cell viability assay

Cell viability was assessed using the WST-1 assay. Cells were pretreated with different concentrations of CK for 24 h and then exposed to various concentrations of TRAIL for 24 h. The medium was then removed, and 10 μl per well of the WST-1 solution (Beyotime Biotechnology, Jiangsu, China) was added. After 4-h incubation, the absorbance was measured at 450 nm using a microplate reader (Bio-Rad, Hercules, CA, USA). All experiments were performed in triplicate and repeated at least three times. Cell viability is expressed as the percentage of the control, which was set to 100%.

### Crystal violet assay

For the crystal violet assay, the cells were treated with CK for 24 h and then treated with a combination of CK and TRAIL for 24 h. The cells were washed with phosphate-buffered saline (PBS), fixed with methanol, stained with crystal violet and then imaged.

### PI staining for DNA fragmentation

Using flow cytometry, cell death was quantified with propidium iodide (PI) staining for DNA fragmentation and content. For this analysis, the cells were pretreated with CK for 24 h and then exposed to TRAIL for 24 h. Floating and adherent cells were collected and fixed in 75% ethanol, followed by RNase A treatment and PI staining. A total of 20 000 fluorescent events were acquired at 610 nm following excitation at 488 nm.

### Reverse transcription polymerase chain reaction (RT-PCR)

DR5 mRNA was detected using RT-PCR as follows: total RNA from the treated cells was extracted using the Trizol reagent according to the supplier's instructions. Two micrograms of total RNA was converted into cDNA using M-MLV reverse transcriptase (Promega, Madison, WI, USA) and then amplified using Ex-Taq polymerase (Takara, Otsu, Shiga, Japan). Total RNA was amplified by PCR with primers described previously: DR4 sense 5′-CTGAGCAACGCAGACTCGCTGTCCAC-3′ and DR4 anti-sense 5′-TCCAAGGAC ACGGCAGAGCCTGTGCCAT-3′ DR5 sense 5′-AAGACCCTTGTGCTCGTTGTC-3′ and DR5 anti-sense 5′-GACACATTCGATGTCACTCCA-3′ glyceraldehyde-3-phosphate dehydrogenase (GAPDH) sense, 5′-GTCTTCACCACCATGGAG-3′ and GAPDH anti-sense 5′-CCACCCTGTTGCTGTAGC-3′. The reactions were run at 94 °C for 2 min; 94 °C for 35 cycles of 30 s each; 50 °C for 30 s; and 72 °C for 45 s with an extension at 72 °C for 10 min. PCR products were run on a 1.5% agarose gel and then stained with ethidium bromide. The stained bands were visualized under UV light and photographed.

### Flow cytometric analysis of the expression of cell surface DR4 and DR5

Cells were treated with different concentrations of CK for 24 h, then cells were collected, washed with cold 2% FBS/PBS and labeled with anti-DR4 or DR5 antibody for 20 min on ice. Secondary antibody used was phycoerythrin-conjugated goat anti-mouse or rabbit, respectively incubated for 20 min on ice, protection from light. Cells were then washed in cold 2% FBS/PBS and resuspended in 300 *μ*l 2% FBS/PBS and immediately for flow cytometry analysis. All the antibobies were diluted with 2% FBS/PBS.

### siRNA transfection

Transfection of HCT116 cells was conducted with Lipofectamine RNAiMAX (GenePharma, Shanghai, China) following the manufacturer's instructions. High purity controls (scrambled RNA), along with DR5, p53, CHOP, Atg7 and JNK siRNA oligos, were obtained from GenePharma. The targeting sequences of the siRNA constructs are: DR5 siRNA-1, 5′-UUCUGGGAACACGAGCAACAG-3′ DR5 siRNA-2, 5′-UUUAGCCACCUUUAUCUCAUUGUCC-3′ p53 siRNA-1, 5′-AAGACUCCAGUGGUAAUCUAC-3′ p53 siRNA-2, 5′-CGGCAUGAACCGGAGGCCCAU-3′ CHOP siRNA-1, 5′-UUCUUGGUCGUCUCCAGUGUU-3′ CHOP siRNA-2, 5′-GCCUGGUAUGAGGACCUGC-3′ Atg7 siRNA-1, 5′-GGAGUCACAGCUCUUCCUU-3′ Atg7 siRNA-2, 5′-GGAACACUGUAUAACACCA-3′ JNK siRNA-1 is a combination of two sequences including 5′-UUCGGGUCAUUAUAUCAUCAU-3′ and 5′-GGCACUUGAGCUGGUGAAUUU-3′ JNK siRNA-2, 5′-UUUUUCUUACAGGAUGGAAGA-3′.

### Western blotting

The cells were lysed in lysis buffer (50 mM Tris/acetate, pH 7.4, 1 mM EDTA, 0.5% Triton X-100, 150 mM sodium chloride, 0.1 mM PMSF, and Roche incomplete protease inhibitor cocktail). The protein concentrations were measured according to the Bradford method.^46^ Equal amounts of protein were separated by 12% sodium dodecyl sulfate-polyacrylamide gel electrophoresis, transferred to a PVDF membrane, blotted with each antibody and detected by using the enhanced chemiluminescence (ECL) reagent (GE Healthcare).

### Measurement of reactive oxygen species

Intracellular ROS were detected by treating the cells with 20 *μ*M dichlorofluorescein diacetate (DCFH-DA) before the end of treatment (20 min), and the increase in fluorescence was measured by flow cytometry.

### Statistical analysis

The data were analyzed using a Student's *t*-test for comparison between groups. Significance was defined as *P*-values <0.05 or 0.01.

## Abbreviations

CHOP: C/EBP homologous protein
CK: compound K
ERKs: extracellular signal-regulated kinases
cFLIP: Fas-associated death domain-like IL-1-converting enzyme-inhibitory protein
DMEM/F-12: Dulbecco's modified Eagle's medium: nutrient mixture F-12
DR: death receptors
DCFH-DA: dichlorofluorescein diacetate
DPI: diphenyleneiodonium chloride
JNK: c-Jun NH2-terminal kinase
IAP: inhibitor of apoptosis protein
HUVECs: human umbilical vein endothelial cells
MAPKs: mitogen-activated protein kinases
NAC: *N*-acetylcysteine
PI: propidium iodide
ROS: reactive oxygen species
PMSF: phenylmethylsulfonyl fluoride
RT-PCR: reverse transcription polymerase chain reaction
TRAIL: tumor necrosis factor (TNF)-related apoptosis-inducing ligand
XIAP: X-linked inhibitor of apoptosis protein
3-MA: 3-methyladenine
