# Supplementary figures and images for: Ginsenoside compound K sensitizes human colon cancer cells to TRAIL-induced apoptosis via autophagy-dependent and -independent DR5 upregulation
Source: Cell Death Dis. 2016 Aug 11;7(8):e2334–. doi: 10.1038/cddis.2016.234 (PMC5108320; doi:10.1038/cddis.2016.234)

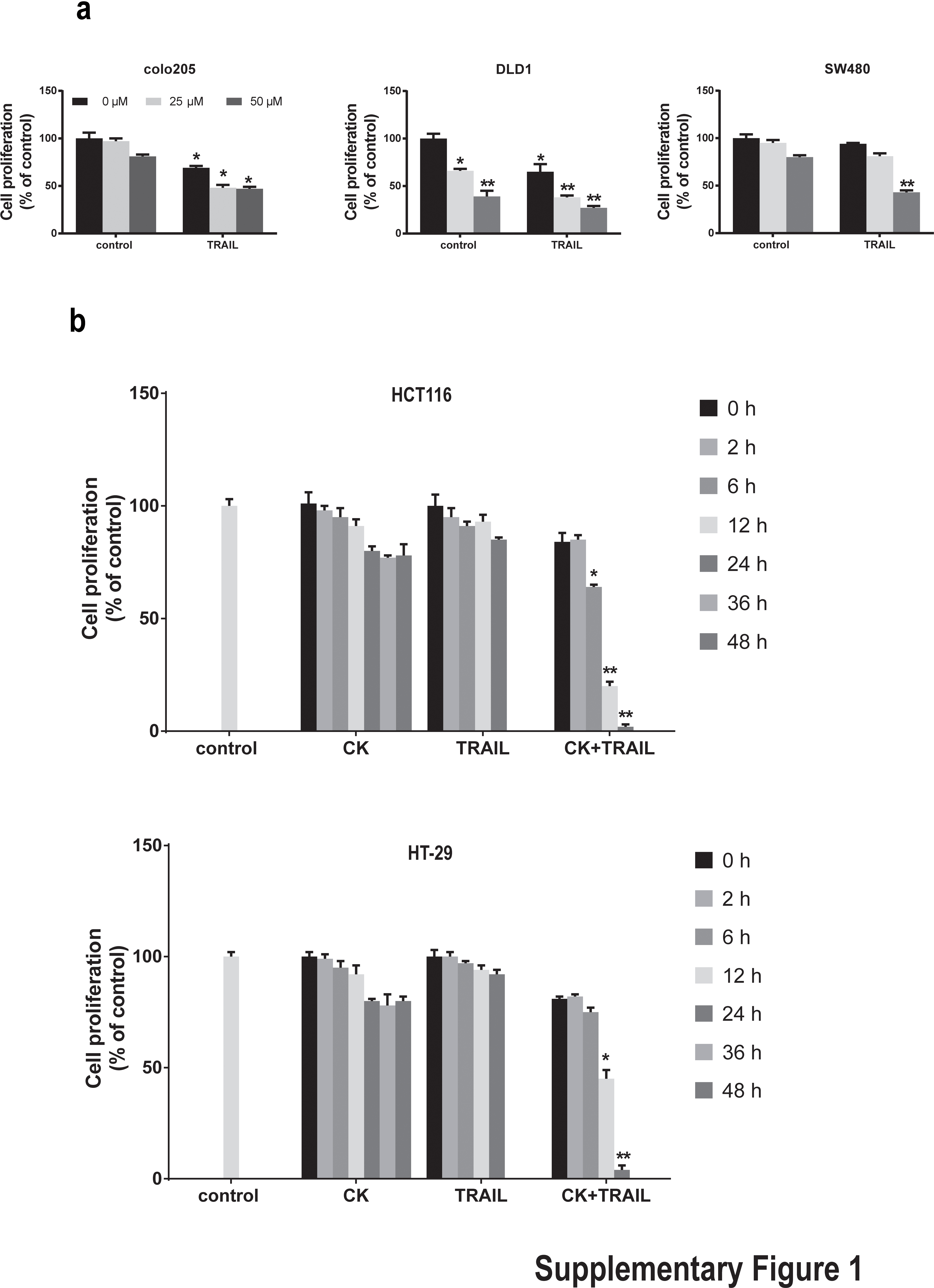

Supplement: Supplementary Figure 1 [file cddis2016234x1.tif]

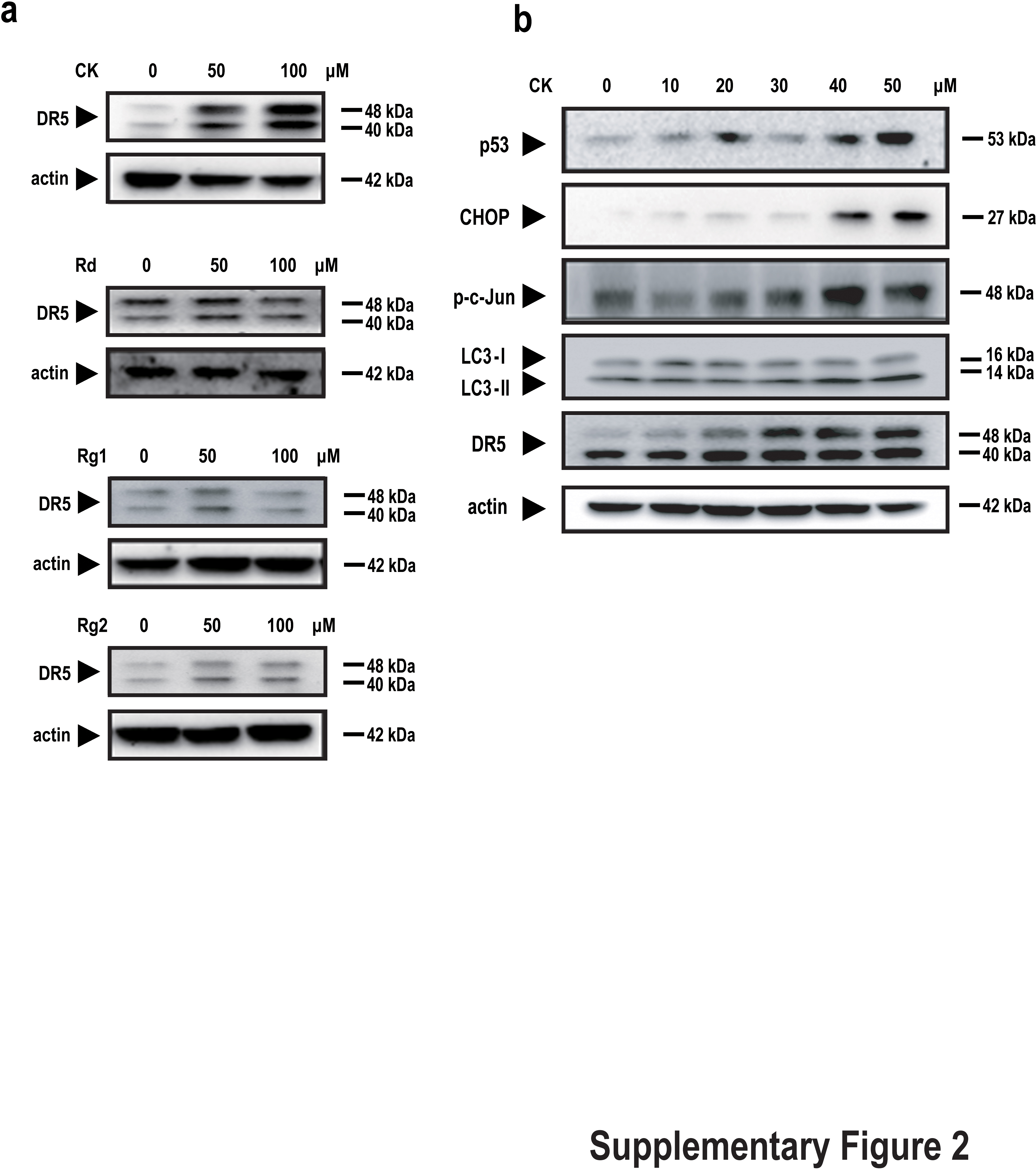

Supplement: Supplementary Figure 2 [file cddis2016234x2.tif]

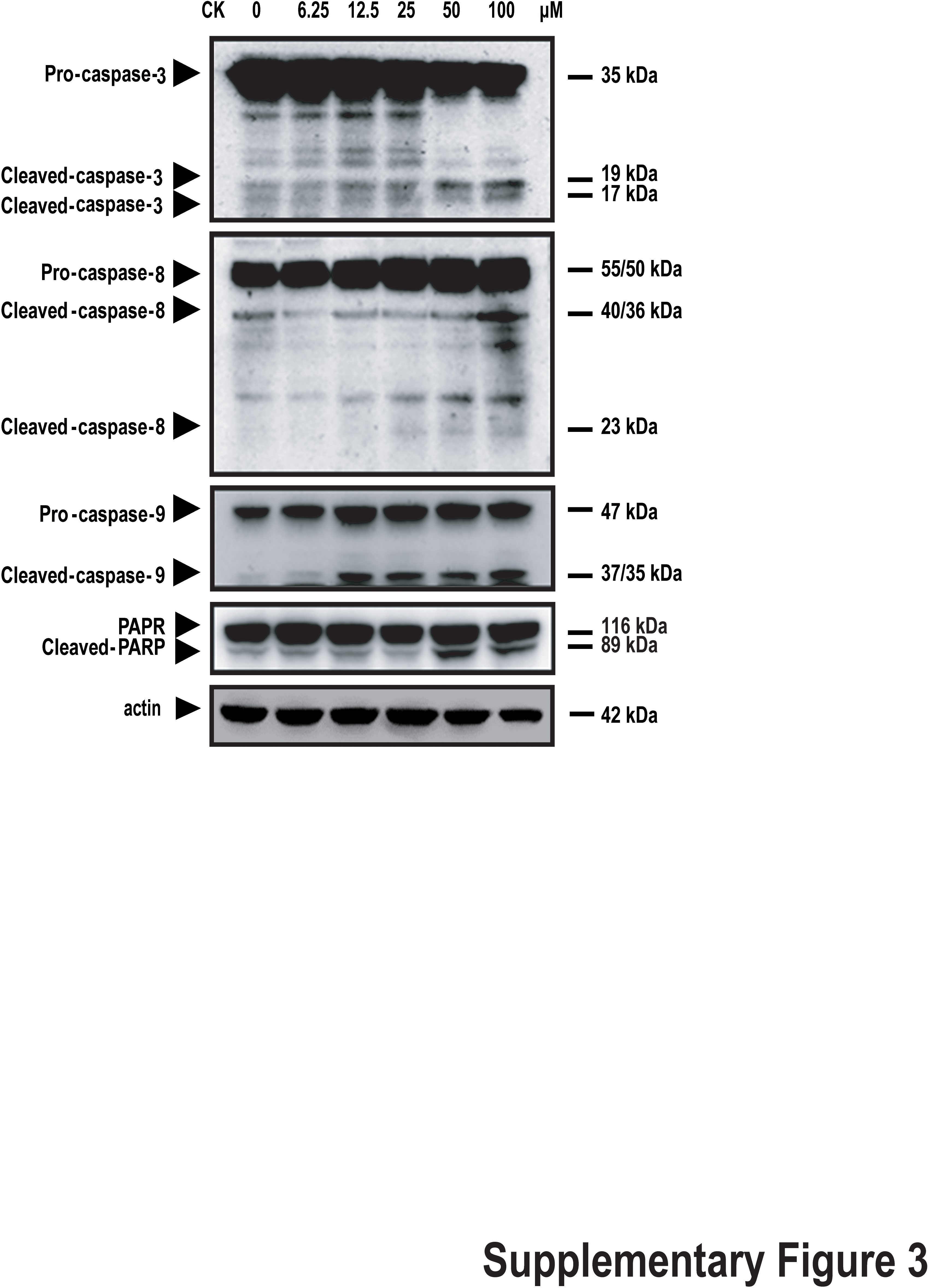

Supplement: Supplementary Figure 3 [file cddis2016234x3.tif]

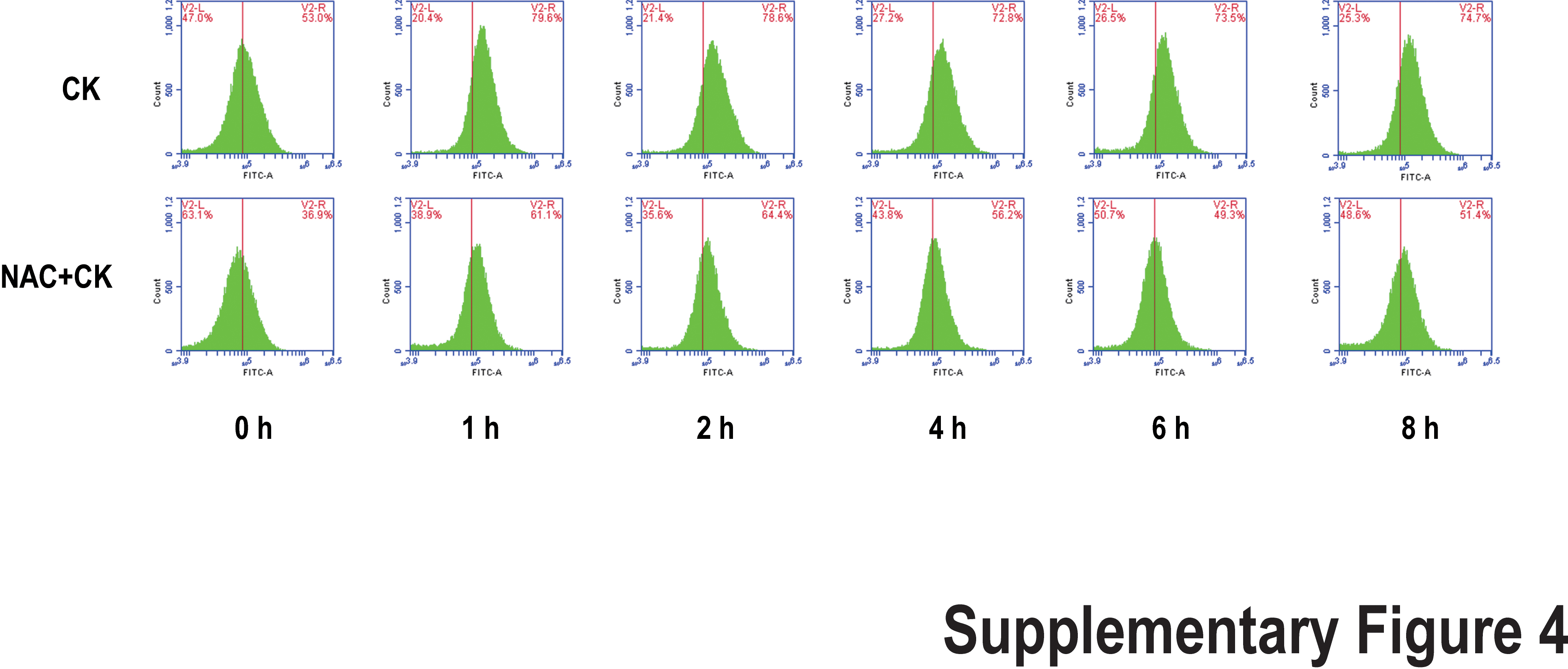

Supplement: Supplementary Figure 4 [file cddis2016234x4.tif]
