# Supplementary material for: Ginsenoside compound K sensitizes human colon cancer cells to TRAIL-induced apoptosis via autophagy-dependent and -independent DR5 upregulation
Source: Cell Death Dis. 2016 Aug 11;7(8):e2334–. doi: 10.1038/cddis.2016.234 (PMC5108320; doi:10.1038/cddis.2016.234)
Supplement: Supplementary Figure Legends [file cddis2016234x5.doc]

**Supplementary Figure Legends**

**Supplementary Fig. 1**. CK enhanced TRAIL-induced apoptosis in Colo205**,** DLD1 and SW480 human colon carcinoma cells. Colo205, DLD1 and SW480 cells were pretreated with or without the 50 μM of CK for 24 h and then co-treated with or without 25 ng/ml of TRAIL for indicated times. Cell viability was analyzed using WST-1 assay.

**Supplementary Fig. 2**. DR5 expression was not impacted by ginsenoside Rd, Rg1 and Rg2 treatment. HCT116 cells were treated with indicated ginsenosides at the indicated doses. DR5 protein was analyzed by western blotting. Actin was used as a protein loading control**.**

**Supplementary Fig. 3**. CK impacted the DR5, p53, CHOP, LC3-II and p-c-Jun expressions at intermediate concentrations. HCT116 cells were pretreated with or without the indicated concentrations of CK, indicated proteins were analyzed by western blotting. Actin was used as a protein loading control**.**

**Supplementary Fig. 4**. Pretreatment with NAC for 1 h abrogated CK induced ROS production. HCT116 cells were pretreated with or without 40 mM NAC for 1 h and then treated with or without CK for indicated times. ROS levels were assessed by flow cytometry followed by DCFH-DA staining.
